# Sex Differences in the Association between Social Support and Major Depression: A Mediation Analysis with Interoception Mediator

**DOI:** 10.31083/AP38763

**Published:** 2025-02-28

**Authors:** Yuqing Wu, Meichen Lu, Xiaohong Liu, Yifan Sun, Zhenhe Zhou, Hongliang Zhou

**Affiliations:** ^1^Department of Psychiatry, The Affiliated Mental Health Center of Jiangnan University, 214151 Wuxi, Jiangsu, China; ^2^Department of Psychiatry, The Affiliated Wuxi Mental Health Center of Nanjing Medical University, 214151 Wuxi, Jiangsu, China; ^3^Department of Psychology, The Affiliated Hospital of Jiangnan University, 214151 Wuxi, Jiangsu, China

**Keywords:** sex difference, major depression, mediation analysis, social support, interoception

## Abstract

**Background:**

Social support is recognized as a critical factor in both the prevention and management of Major depression Disorder (MDD), and can influence interoceptive processes. The mechanism of sex differences in the association between social support and MDD has not been clarified. This study was to elucidate the mechanism of sex differences in the association between social support and MDD by a mediation analysis with interoception mediator.

**Methods:**

Participants included 390 depressed patients (male/female: 150/240). Social Support Rating Scale (SSRS) was used to assess the degree of social support; Multidimensional Assessment of Interoceptive Awareness (MAIA-2C) was used to evaluate the interoception; Patient Health Questionnaire-9 (PHQ-9) was used to assess depression status. The pairwise correlated variables were put into the mediation model for the mediation analysis.

**Results:**

The depression status in female depressed patients was more severity than that in male depressed patients, while the social support in female depressed patients was less than that in male depressed patients. In male depressed patients, the Noticing of MAIA-2C plays a partial mediating role in social support and depression status, however, in female depressed patients, the Self-Regulation and Trusting of MAIA-2C plays a partial mediating role in social support and depression status.

**Conclusions:**

The female depressed patients receive significantly less social support than male counterparts, contributing to more severe symptoms, with the quality and adequacy of social support being crucial due to its mediation by interoception, highlighting a biological mechanism behind MDD. Differences in how interoception mediating role between genders suggest a physiological reason for the heightened severity of depressive symptoms in females.

## Main Points

1. This is the first to investigate the mechanism of sex differences in the association between Social support and Major depression with a large sample.

2. The female depressed patients receive significantly less Social support due to its mediation by interoception, highlighting a biological mechanism behind Major depression.

3. Differences in how interoception mediating role between sexes suggest a physiological reason for the heightened severity of depressive symptoms in females.

## 1. Introduction

Major Depressive Disorder (MDD) is one of the most common mental disorders worldwide. It presents as a persistent state of pervasive sadness, a marked decrease in pleasure or interest in most activities, or fatigue or loss of energy almost every day [1, 2]. The etiology of MDD is multifactorial, involving a complex interplay of genetic, biological, environmental, and psychological factors [3]. Although MDD can affect individuals of all genders, substantial evidence suggests variations in its manifestation and impact between men and women [4]. Epidemiological studies consistently report a higher prevalence of MDD among women [5]. Various factors contribute to this phenomenon, including hormonal fluctuations, psychosocial stressors [6], and differences in help-seeking behaviors [7]. Sex differences in the symptomatology of MDD have been observed, with women more likely to report symptoms such as feelings of worthlessness, guilt, and somatic complaints [8]. These differences may stem from biological, psychological, and sociocultural factors, highlighting the need for a nuanced understanding of symptom presentation in clinical assessment and diagnosis. Research has suggested that sex-specific vulnerabilities, such as hormonal fluctuations during reproductive stages, may contribute to differential susceptibility to MDD [9]. Additionally, psychosocial stressors, including sex-based discrimination and interpersonal relationships, may play a role in shaping sex differences in MDD risk [10]. Based on above outcome, Kuehner [11] suggested that an integration of the research domain criteria framework will allow examination of gender differences in core psychological functions, within the context of developmental transitions and environmental settings.

### 1.1 Social Support and Depression in Sex Differences

Social support refers to the psychological and material resources provided by family, friends, healthcare professionals, and social networks, which are intended to benefit an individual’s ability to cope with stress [12]. It encompasses emotional support (empathy, love, trust), instrumental support (tangible aid and services), informational support (advice, guidance), and appraisal support (affirmation) [13]. Sociologically, empirical studies have indicated that women and men tend to experience and utilise social support differently due to diverse factors such as socialisation processes, gender norms, and societal expectations [14, 15, 16, 17]. Women are generally more likely to seek and offer emotional support within their social networks than do men [18]. Men are more inclined towards instrumental support, reflecting societal expectations of male independence and stoicism [19]. The influence of cultural norms and values cannot be understated in shaping the nature of social support between sexes [20]. In societies in which traditional sex roles are strongly upheld, the divergence in social support types and networks between men and women is more marked. Conversely, in more egalitarian societies, where sex roles are less rigidly defined, there is a tendency towards a more balanced distribution of emotional and instrumental support among genders. Furthermore, the interaction of sex with other social categories such as race, class, and age introduces additional complexity into the understanding of social support dynamics [21, 22].

Previous studies indicated that social support affects mental and physical health indirectly or directly through the stress buffering model and the main effect hypothesis [23, 24]. The stress buffering hypothesis posits that an individual’s social resources can prevent or mitigate the impact of stress on health. Social resources can intervene in the pathway from stress to disease by attenuating the stress appraisal response or reducing the stress reaction [23, 24]. Social support can lead to changes in physiological processes such as cardiovascular, endocrine, and immune systems, becoming a potential mechanism of depression [12]. Furthermore, the main effect model of social support emphasizes the intrinsic value of social support as a resource in itself, rather than merely providing assistance in stressful situations [25]. Social support can also improve depression in women of childbearing age [26]. In addition, there are gender differences between social support and depression, with women more sensitive to the depressive effects of low social support than men [27]. Social support is widely recognized as a critical factor in both the prevention and management of MDD [28, 29, 30]. Social support can act as a buffer against the stressors that often precipitate or exacerbate depressive symptoms [31, 32, 33]. For individuals already suffering from MDD, social support is vital in the treatment and recovery process [34]. Additionally, research has also shown that perceived social support, more than actual received support, is crucial in influencing mental health outcomes [35]. Social support acts as a protective factor against the severity and duration of depressive episodes. Many studies have reported that sex plays a crucial role in shaping the social support experiences of individuals with MDD [36, 37, 38]. The disparities in perceived and received social support between male and female depressed patients underscore the need for gender-sensitive approaches in mental health care and support systems. However, the mechanisms underlying these sex differences have been unclear.

### 1.2 Interoception and Depression in Sex Differences

Interoception refers to the process by which the nervous system senses, interprets, and integrates signals originating from within the body, such as heart rate, respiratory rate, hunger, thirst, and the sensation of internal organ activity [39, 40]. Interoceptive signals originate from receptors inside the body, including organs, muscles, and skin, which relay information about the body’s internal state to the brain, primarily to the insular cortex [41]. The interpretation of bodily signals contributes to the subjective experience of emotions [42]. In addition, interoception is associated with various medical and psychological conditions, including anxiety disorders, depression, eating disorders, and chronic pain syndromes [43]. Individuals with poor interoceptive awareness may have difficulty recognizing and responding to their own emotional and physical needs [44].

In previous studies, although men have higher interoceptive accuracy than women [45], women have an advantage over men in identifying and processing their own and others’ emotions [46]. Based on this contradictory phenomenon, Robert Kegan, in a study on gender differences in emotional perception, has indicated that men and women rely on different types of cues for measuring internal states and emotional regulation, with men tending to use internal physiological cues and women more inclined to use external environmental cues [47].

The Bayesian Inference Model is a statistical method that allows individuals to continuously update beliefs or hypotheses based on prior knowledge and new evidence [48]. Predictive coding and prediction errors are key components of the Bayesian inference model in the context of interoceptive inference [44, 49, 50, 51]. It has been confirmed that people’s construction of the external world depends on the dynamic balance of the brain’s perception and internal bodily signals, and to maintain homeostasis, the brain generates predictions about the actual state of the body (priors) based on past experiences and the current environment [52]. Interoception perceives, integrates, and interprets bodily signals from any part of the body, obtaining perceptual data (likelihoods). The brain combines prior and likelihood data to calculate, allowing the brain to achieve minimal predictive error, reduce the discrepancy between the predicted state of the world and the actual state, and make corresponding adjustments and bodily preparations in advance, enabling the body to better face complex environments. Within the framework of interoceptive inference theory, emotions are conscious products that arise when the brain actively infers that an error needs to be explained in the interoceptive predictions when the prior is greater than the likelihood, that is, the brain actively seeks the possible causes of bodily changes [52]. Study results have suggested that MDD is a problem of interoceptive dysfunction, and its mechanism involves a mismatch between predictive coding (what the brain expects to perceive) and the actual sensory input; a prediction error occurs [53, 54]. MDD patients often exhibit impaired interoceptive accuracy [55]. This impairment may affect emotional processing and mood regulation. Such disruptions in interoceptive processes are thought to contribute to the hallmark symptoms of MDD, including dysregulated affect and anhedonia (the inability to feel pleasure) [56]. Based on the Bayesian inference model and interoceptive inference theory, interoceptive dysregulation in MDD may stem from abnormalities in the brain’s interoceptive pathways, including the insular cortex, anterior cingulate cortex, and somatosensory cortex [57, 58]. Furthermore, the relationship between interoception and MDD is bidirectional [59]. Not only can impaired interoception contribute to the onset and severity of depressive symptoms, but the chronic stress and emotional dysregulation characteristic of MDD can further disrupt interoceptive signaling, thereby creating a vicious cycle that may exacerbate the disorder.

### 1.3 Social Support and Interoception

Social support can influence interoceptive processes [60], for instance, positive social interactions may enhance interoceptive accuracy by modulating physiological responses to stress and emotional states [61]. Conversely, interoceptive awareness can influence one’s perception and utilisation of social support [62], as individuals with heightened interoceptive sensitivity may be more attuned to their emotional needs and, by extension, more adept at seeking out and utilizing social support in times of distress.

Summarily, social support, interoception, and MDD are interconnected constructs that play significant roles in psychological and emotional well-being. The pairwise relationships in social support, interoception, and MDD are all intricate and bidirectional. As yet, the mechanism of sex difference in the association between social support and MDD has not been clarified. Understanding the relationship among social support, interoception and MDD would be helpful in elucidating the mechanism of social support in the sex differences underlying the prevention of MDD. Further research into the mechanism and the development of targeted interventions can contribute to more equitable and effective support for all individuals suffering from MDD.

In the present study, we conduct a mediation analysis based on the Biopsychosocial Model to construct a mediation model [63, 64]. The Social Support Rating Scale (SSRS) was used to investigate social support [65], the Multidimensional Assessment of Interoceptive Awareness- 2nd Edition, Chinese version (MAIA-2C) scale was used to assess interoception [66], and the Patient Health Questionnaire-9 (PHQ-9) was used to assess depression status [67]. A mediation analysis with interoception mediator was conducted in order to explore the association between social support and MDD in the depressed samples. Based on the previous studies [45, 46, 60], Our hypothesis was that: (1) inadequate or the low-quality social support leads to MDD through interoception mediating; (2) there are sex differences in the way that reduced social support leads to MDD through interoception; (3) depressed female patients are more sensitive to this pathway. The purpose of the present study was to elucidate the mechanism of sex differences in the association between social support and MDD by a mediation analysis with interoception mediator.

## 2. Materials and Methods

### 2.1 Study Sample

A total of 390 depressed patients (male/female: 150/240) were included in this study. All patients were from the Department of Clinical Psychology of the Affiliated Mental Health Centre of Jiangnan University, Jiangsu Province, China. The study was conducted from June 1, 2022 to December 31, 2023. Inclusion criteria: (1) meet the diagnostic criteria for MDD in the Statistical Diagnostic Manual of Mental Disorders (DSM-5) of the American Psychiatric Association; (2) 18–65 years old; (3) emotionally stable and cooperative; (4) provided informed consent (self or guardian). Exclusion criteria: (1) cerebral organ disease or serious unstable physical disease (such as coronary heart disease or diabetes); (2) current or recent serious suicide attempt or behaviors; (3) Young’s Mania Rating Scale (YMRS) scores of more than 5.

### 2.2 Demographic Measurement

Sociodemographic data included sex, age, height, weight, body mass index (BMI), years of education, marital status (single, married, divorced), birth/raise status (childless, having one child, having two or more children), and total household annual income.

### 2.3 Social Support Assessment

The SSRS was used to assess the degree of social support of each patient [68]. There are 10 items and three dimensions in the scale, and the Cronbach’s α coefficients of the items and total scores ranged from 0.825 to 0.896, with good reliability and validity [69]. The three dimensions are divided into Objective Support, Subjective Support, and Support Utilisation. Objective Support refers to objective, visible, or practical support; Subjective Support refers to the personal emotional experience of being respected, supported, and understood in the community; Support Utilisation refers to the extent to which social support is used.

### 2.4 Evaluation of Interoception

The MAIA-2C was used to evaluate interoception [66]. The Cronbach’s α of the total scale was 0.822, and the sub-scales ranged from 0.656 to 0.838, with good reliability and validity [66]. The scale has 37 items and consists of the following eight subscales: (1) Noticing — awareness of uncomfortable, neutral, or comfortable physical sensations; (2) Not Distracting — the tendency to ignore or distract oneself from sensations such as pain or discomfort; (3) Not Worrying — emotional distress or worry about feelings of pain or discomfort; (4) Attention Regulation — the ability to control attention to bodily sensations; (5) Emotional Awareness — awareness of the connection between physical sensations and emotional states; (6) Self-Regulation — the ability to regulate the perception of pain by paying attention to physical sensations; (7) Body Listening — how a person actively attends to the body to gain insight; (8) Trusting — the physical experience of being safe and trustworthy.

### 2.5 Assessment of Depression Status 

The PHQ-9, developed based on the nine-symptom criteria for the diagnosis of MDD in the DSM-5, was used to assess depression status [70]. It has good reliability and validity; Cronbach’s α >0.747 [71, 72]. The questionnaire uses a 4-point scale to rate the severity of depressive symptoms over the previous two weeks. If the symptom corresponding to the question did not appear in the previous two weeks, the assigned score was 0; the symptom score was 1 for a few days, 3 for more than half the time, and 4 for almost every day. The total number of points ranges from 0 to 27, with higher scores indicating more severe depression.

### 2.6 Statistical Analysis

Data were recorded in Microsoft Excel 2016, Version 15.0, developed by Microsoft Corporation, Redmond, WA, USA. and analyzed with the Statistical Package Statistics Software version 24.0 (SPSS 24.0, Inc., Chicago, IL, USA). Clinical data were compared by *t*-test for continuous, normal distribution, independent quantitative variables, and compared by rank-sum test for the non-normal distribution, quantitative variables. Chi-square analysis was used to determine the relationship between qualitative variables. Pearson correlation analysis (Bonferroni modified method) was used for pairwise correlation analysis of the MAIA-2C total scores and subscale scores, SSRS total scores and dimension scores, and PHQ-9 scores. The jamovi 2.3.28 software was used for mediation analysis. Pairwise correlated variables (the MAIA-2C total scores and subscale scores, SSRS total scores and dimension scores, and PHQ-9 scores) were put into the mediation model, and Boostrap (5000) was used for validity verification.

## 3. Results

### 3.1 Analysis of Demographic Data and the SSRS, MAIA-2C, PHQ-9 Scores

Demographic data are shown in Table 1. There was no significant difference in age, years of education, marriage, birth/raise status, total family annual income, body weight or BMI between male and female MDD patients.

**Table 1.** **Demographic data and the SSRS, MAIA-2C, PHQ-9 scores**.

Variable		Male (n = 150)	Female (n = 230)	Test statistic
Age (years), M (P25, P75)		22.00 (18.00, 32.25)	22.00 (18.00, 32.25)	z = –0.04, *p* = 0.961
Height (centimeter), M (P25, P75)		174.50 (170.00, 178.00)	163.00 (160.00, 168.00)	z = –13.61, *p * < 0.001
Weight (kilogram), M (P25, P75)		68.00 (57.00, 80.00)	54.00 (47.00, 62.12)	z = –9.19, *p * < 0.001
Education (years), M (P25, P75)		12.00 (11.00, 15.25)	13.00 (11.00, 15.00)	z = –0.61, *p* = 0.544
Marriage Status, N (%)				
	Single	112 (74.7%)	159 (69.1%)	χ^2^ = 2.01, *p *= 0.365
	Married	36 (24.0%)	64 (27.8%)	
	Divorced	2 (1.3%)	7 (3.0%)	
Birth/Raise Status, N (%)				
	Childless	116 (77.3%)	166 (72.2%)	χ^2^ = 1.35, *p* = 0.507
	One child	29 (19.3%)	53 (23.0%)	
	Two children or more	5 (3.3%)	11 (4.8%)	
Total Household Annual Income (RMB), N (%)				
	<¥100,000	38 (25.3%)	51 (22.9%)	χ^2^ = 1.20, *p* = 0.752
	¥100,000 – ¥300,000	96 (64.0%)	147 (64.2%)	
	¥300,000 – ¥600,000	13 (8.7%)	25 (10.1%)	
	>¥600,000	3 (2.0%)	7 (2.8%)	
BMI, M (P25, P75)		22.41 (18.58, 25.80)	19.77 (17.93, 23.14)	z = –4.35, *p * < 0.001
Mania, M (SD)		2.27 (1.32)	1.80 (1.26)	*t *= 3.41, *p* = 0.001
PHQ-9, M (P25, P75)		18.00 (14.00, 21.25)	19.00 (15.00, 23.00)	z = –2.58, *p* = 0.010
MAIA-2C (and subscales)				
	Total	16.95 (4.00)	16.59 (3.92)	*t *= 0.86, *p* = 0.389
	Noticing, Mean (SD)	2.84 (0.94)	2.77 (0.98)	*t* = 0.62, *p* = 0.536
	Not Distracting, Mean (SD)	2.68 (0.98)	2.77 (1.06)	*t* = –0.83, *p* = 0.406
	Not Worrying, Mean (SD)	2.01 (0.91)	2.09 (0.96)	*t* = –0.81, *p* = 0.416
	Attention Regulation, Mean (SD)	2.09 (0.87)	1.99 (0.92)	*t* = 1.09, *p* = 0.274
	Emotional Awareness, Mean (SD)	2.53 (1.03)	2.49 (1.10)	*t* = 0.33, *p* = 0.735
	Self-Regulation, M (P25, P75)	1.25 (0.75, 1.75)	1.00 (0.50, 1.75)	z = –1.79, *p* = 0.073
	Body Listening, M (P25, P75)	1.50 (1.00, 2.33)	1.33 (0.67, 2.33)	z = –0.73, *p* = 0.461
	Trusting, M (P25, P75)	1.67 (1.00, 2.33)	1.67 (1.00, 2.33)	z = –0.77, *p* = 0.440
Social Support (and subscales), M (SD)				
	Total	38.42 (10.05)	30.67 (7.19)	*t* = 8.74, *p * < 0.001
	Objective Support	8.64 (3.08)	8.89 (3.02)	*t* = –0.77, *p* = 0.441
	Subjective Support	16.52 (4.57)	15.90 (4.31)	*t *= 1.34, *p* = 0.179
	Support Utilization	5.70 (1.57)	5.89 (1.51)	*t *= –1.18, *p* = 0.237

M (SD), Mean (Standard Deviation). M (P25, P75), Median and interquartile range (25th percentile, p25, and 75th percentile, p75). N (%), Numbers percent of the total. The current exchange rate of RMB to USD is 1 USD = 6.95 RMB. SSRS, Social Support Rating Scale; MAIA-2C, Multidimensional Assessment of Interoceptive Awareness- 2nd Edition, Chinese version; PHQ-9, Patient Health Questionnaire-9.

The SSRS total scores and dimension scores, MAIA-2C total scores and subscale scores, and PHQ-9 scores are shown in Table 1. There were no significant sex differences in the SSRS dimension scores, or the MAIA-2C total scores or subscale scores. However, there were significant differences in the SSRS total scores, and PHQ-9 scores between male and female depressed patients. The depression status in female depressed patients was more severe than that in male depressed patients, and the social support in female depressed patients was less than that in male depressed patients.

### 3.2 Pairwise Correlation Analysis of the SSRS, MAIA-2C, PHQ-9 Scores

**Fig. 1.**
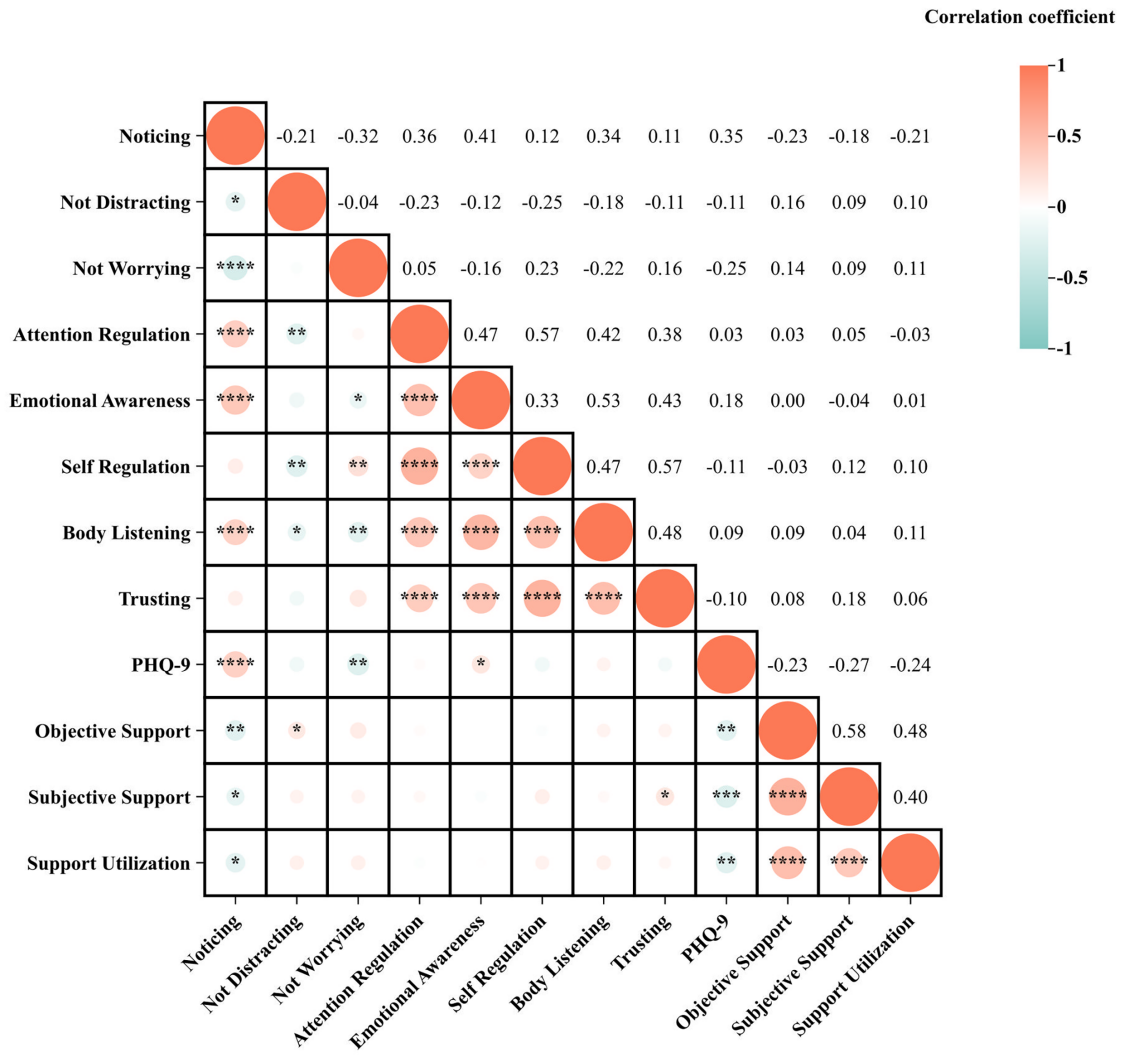
**Pearson’s correlation matrix of male depressed patients**. *p < 0.05; **p < 0.01; *** *p* < 0.001; **** *p* < 0.0001.

**Fig. 2.**
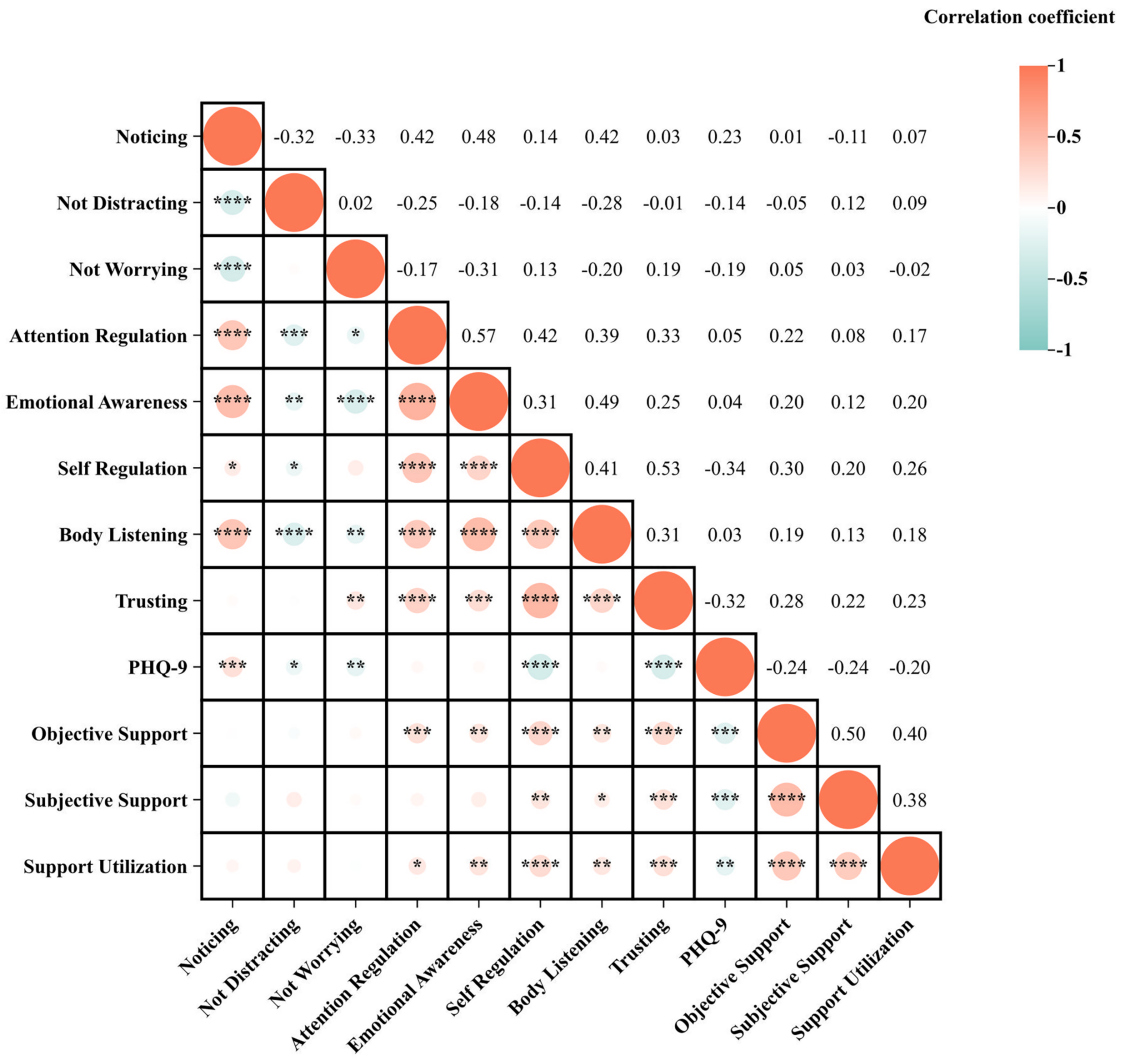
**Pearson’s correlation matrix of female depressed patients**. *p < 0.05; **p < 0.01; *** *p* < 0.001; **** *p* < 0.0001.

**Fig. 3.**
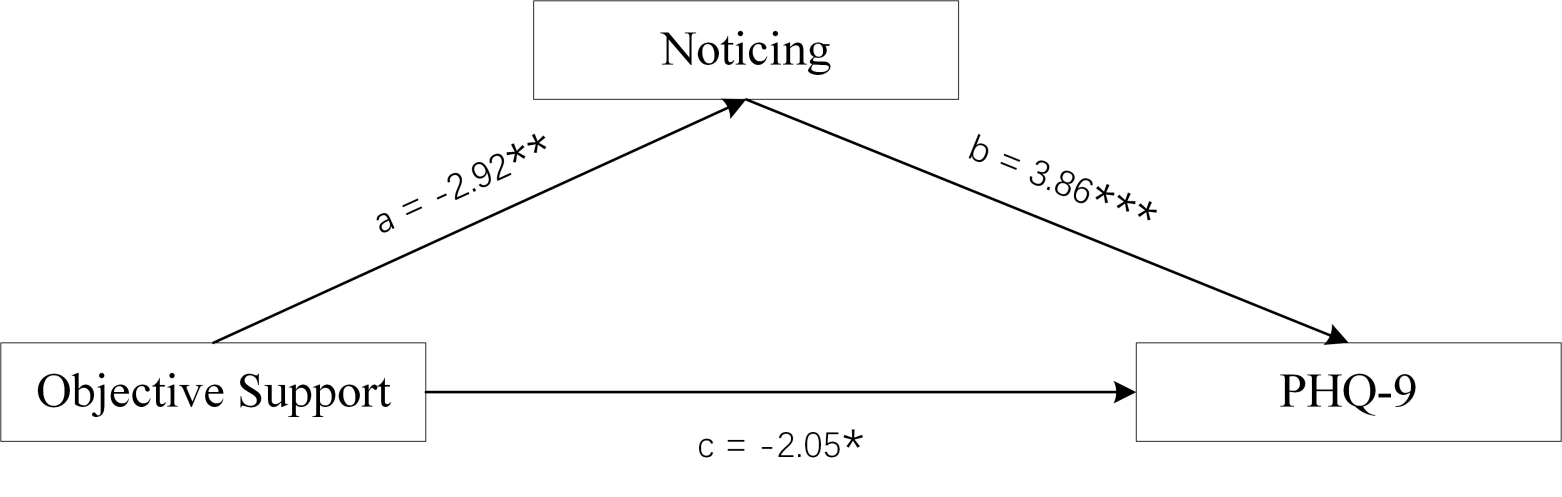
**Mediation Model of male group**. Objective Support was used as independent variables, PHQ-9 scores as dependent variable, and Noticing scores of MAIA-2C as mediating variable. *p < 0.05; **p < 0.01; ***p < 0.001.

**Fig. 4.**
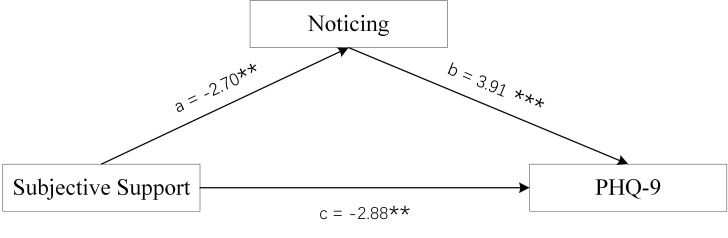
**Mediation Model of male group**. Subjective Support was used as independent variables, PHQ-9 scores as dependent variable, and Noticing scores of MAIA-2C as mediating variable. **p < 0.01; ***p < 0.001.

**Fig. 5.**
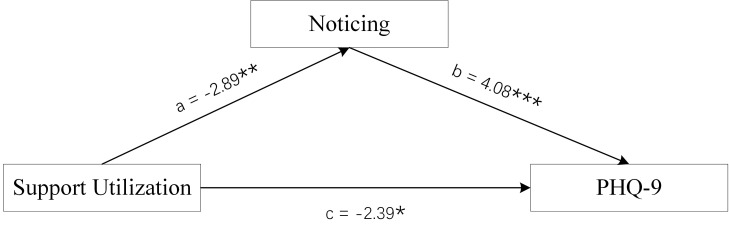
**Mediation Model of male group**. Support Utilization was used as independent variables, PHQ-9 scores as dependent variable, and Noticing scores of MAIA-2C as mediating variable. *p < 0.05; **p < 0.01; ***p < 0.001.

Figs. 1,2 show the correlation matrix between the MAIA-2C total scores and subscale scores, SSRS total scores and dimension scores, and PHQ-9 scores. In male MDD patients, there was a negative correlation between the three dimension scores of the SSRS. Regarding the MAIA-2C, and PHQ-9 scores, there was a positive correlation between the Objective Support dimension scores of SSRS and the Not Distracting scores of MAIA-2C; there was a positive correlation between the Subjective Support scores of SSRS and the Trusting scores of MAIA-2C; there was a positive correlation between PHQ-9 scores and the Noticing scores of MAIA-2C; there was a negative correlation between PHQ-9 scores and the Not Worrying scores of MAIA-2C, and the Emotional Awareness scores of MAIA-2C. The Noticing scores of MAIA-2C may be the mediating variable between Social support and depression status (Figs. 3,4,5).

**Fig. 6.**
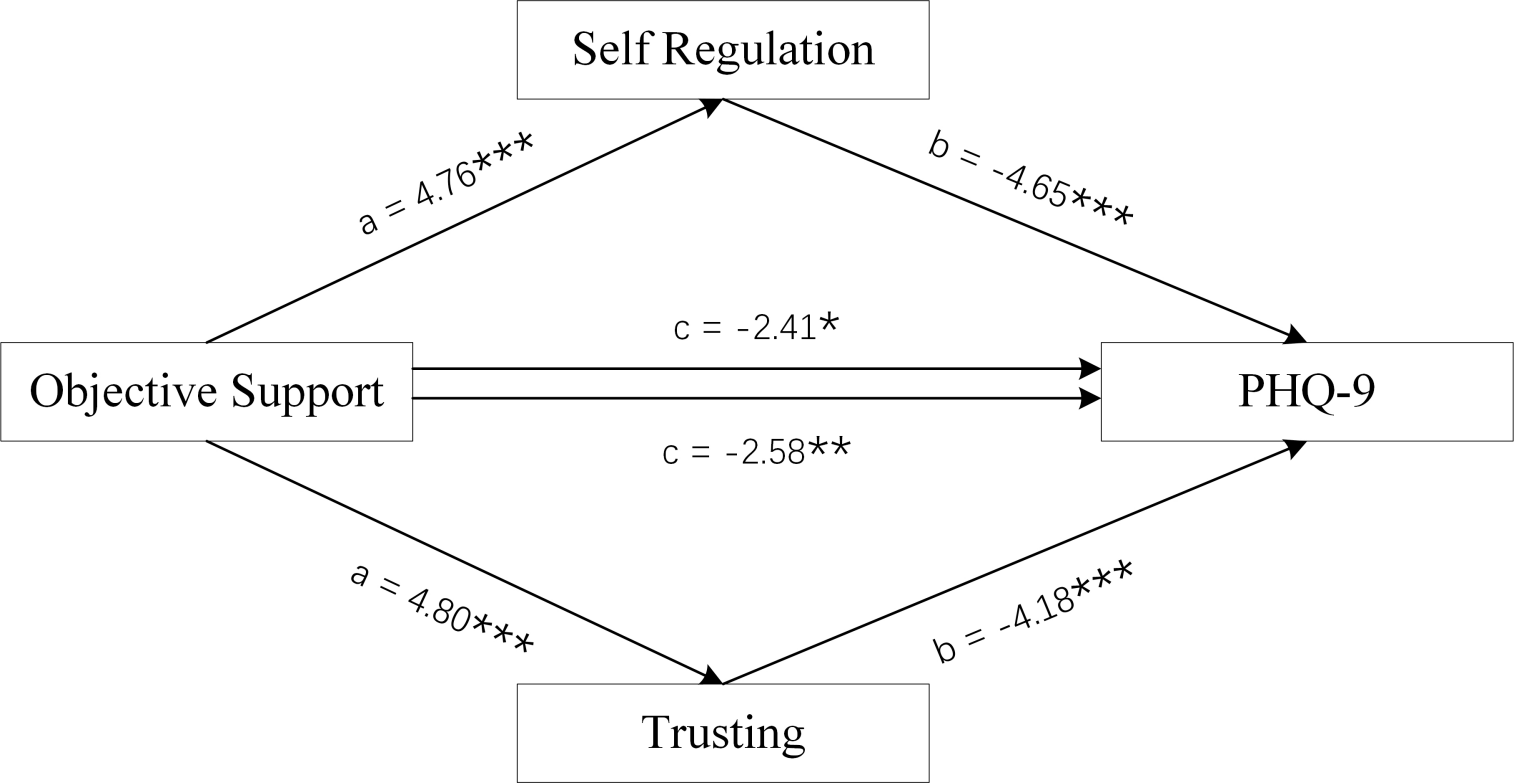
**Mediation Model of female group**. Objective Support was used as independent variables, PHQ-9 scores as dependent variable, and Self-Regulation and Trusting scores of MAIA-2C as mediating variable. *p < 0.05; **p < 0.01; ***p < 0.001.

**Fig. 7.**
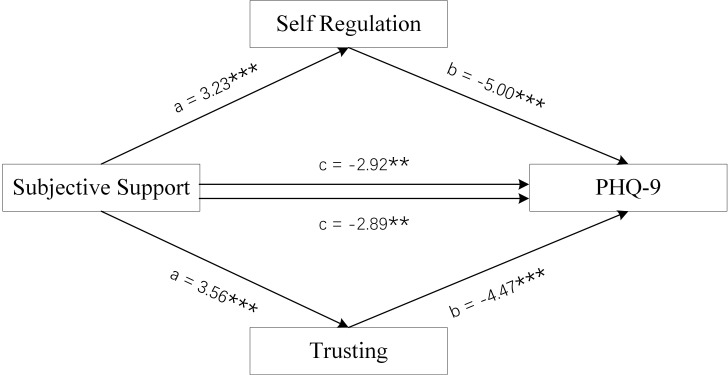
**Mediation Model of female group**. Subjective Support was used as independent variables, PHQ-9 scores as dependent variable, and Self-Regulation and Trusting scores of MAIA-2C as mediating variable. **p < 0.01; ***p < 0.001.

**Fig. 8.**
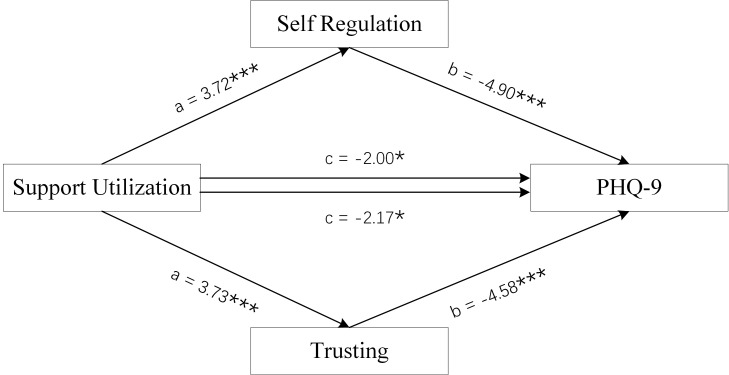
**Mediation Model of female group**. Support Utilization was used as independent variables, PHQ-9 scores as dependent variable, and Self-Regulation and Trusting scores of MAIA-2C as mediating variable. *p < 0.05; *** *p* < 0.001.

In female MDD patients, there was a positive correlation between the three dimension scores of SSRS and the Self-Regulation scores of MAIA-2C, the Body Listening scores of MAIA-2C, and the Trusting scores of MAIA-2C; there was a negative correlation between the three dimension scores of SSRS and PHQ-9 scores; there was a positive correlation between the Objective Support dimension and the Support Utilisation scores, and between the Attention Regulation scores of MAIA-2C and the Emotional Awareness scores of MAIA-2C; there was a positive correlation between PHQ-9 scores and the Attention Regulation scores of MAIA-2C; there was a negative correlation between PHQ-9 scores and the Not Worrying scores of MAIA-2C, the Not Distracting scores of MAIA-2C, the Self-Regulation scores of MAIA-2C, and the Trusting scores of MAIA-2C. There was a pairwise correlation between Social Support, Self-Regulation/Trusting of Interoception, and depression status, suggesting the existence of a mediating effect (Figs. 6,7,8).

### 3.3 Analysis of the Mediating Effect

The Bootstrap method was used to test the mediation model, and the sample size was set to 5000. The three dimension scores of male SSRS scores (Objective Support, Subjective Support, and Support Utilisation) were used as the independent variables, PHQ-9 scores as the dependent variable, and the Noticing scores of MAIA-2C as the mediating variable. The three dimension scores for female SSRS scores (Objective Support, Subjective Support, and Support Utilisation) were used as the independent variables, PHQ-9 scores as the dependent variable, and the Self-Regulation scores and Trusting scores of MAIA-2C as the mediating variables.

Figs. 3,4,5 and Table 2 show the total effect of the model for the analysis of the mediating role of the Noticing scores of MAIA-2C in male MDD patients. All dimension scores of SSRS had a negative direct effect on PHQ-9 scores, and the Noticing scores of MAIA-2C had an indirect effect on PHQ-9 scores, suggesting that the Noticing dimension of MAIA-2C plays a partial mediating role in Social Support and depression status.

**Table 2.** **The total effect of the model for the analysis of the mediating role of Noticing scores of MAIA-2C in male depressed patients**.

Type	Effect	Estimate	SE	95% CI		z	*p*
				Lower	Upper		
Indirect Effect	Objective Support → Noticing → PHQ-9	–0.108	0.048	–0.215	–0.025	–2.22	0.026
	Subjective Support → Noticing → PHQ-9	–0.057	0.026	–0.116	–0.011	–2.13	0.033
	Support Utilization → Noticing → PHQ-9	–0.194	0.086	–0.386	–0.047	–2.24	0.025
Direct Effect	Objective Support → PHQ-9	–0.245	0.119	–0.472	–0.007	–2.05	0.040
	Subjective Support → PHQ-9	–0.217	0.075	–0.366	–0.071	–2.88	0.004
	Support Utilization → PHQ-9	–0.531	0.221	–0.965	–0.092	–2.39	0.017
Total Effect	Objective Support → PHQ-9	–0.352	0.121	–0.585	–0.102	–2.89	0.004
	Subjective Support → PHQ-9	–0.274	0.075	–0.419	–0.126	–3.66	<0.001
	Support Utilization → PHQ-9	–0.725	0.225	–1.170	–0.273	–3.21	0.001

SE, standard error. 95% CI, 95% Confidence Interval.

Figs. 6,7,8 and Tables 3,4 show the total effect of the model for the analysis of the mediating role of the Self-Regulation and Trusting scores of MAIA-2C in female MDD patients. All dimension scores of SSRS had a negative direct effect on PHQ-9 scores, and the Self-Regulation and Trusting scores of MAIA-2C had an indirect effect on PHQ-9 scores, suggesting that the Self-Regulation and Trusting scores of MAIA-2C play a partial mediating role in social support and depression status.

**Table 3.** **The total effect of the model for the analysis of the mediating role of Self-Regulation of MAIA-2C in male depressed patients**.

Type	Effect	Estimate	SE	95% CI		z	*p*
				Lower	Upper		
Indirect Effect	Objective Support → Self-Regulation → PHQ-9	–0.149	0.044	–0.246	–0.070	–3.31	<0.001
	Subjective Support → Self-Regulation → PHQ-9	–0.070	0.026	–0.131	–0.026	–2.62	0.009
	Support Utilization → Self-Regulation → PHQ-9	–0.268	0.089	–0.469	–0.116	–3.00	0.003
Direct Effect	Objective Support → PHQ-9	–0.259	0.107	–0.468	–0.048	–2.41	0.016
	Subjective Support → PHQ-9	–0.209	0.071	–0.352	–0.073	–2.92	0.004
	Support Utilization → PHQ-9	–0.398	0.199	–0.801	–0.014	–2.00	0.046
Total Effect	Objective Support → PHQ-9	–0.408	0.106	–0.616	–0.197	–3.82	<0.001
	Subjective Support → PHQ-9	–0.279	0.073	–0.427	–0.137	–3.83	<0.001
	Support Utilization → PHQ-9	–0.667	0.200	–1.066	–0.282	–3.33	<0.001

SE, standard error. 95% CI, 95% Confidence Interval.

**Table 4.** **The total effect of the model for the analysis of the mediating role of Trusting scores of MAIA-2C in male depressed patients**.

Type	Effect	Estimate	SE	95% CI		z	*p*
				Lower	Upper		
Indirect Effect	Objective Support → Trusting → PHQ-9	–0.126	0.040	–0.214	–0.056	–3.14	<0.001
	Subjective Support → Trusting → PHQ-9	–0.072	0.024	–0.123	–0.029	–2.95	0.003
	Support Utilization → Trusting → PHQ-9	–0.218	0.082	–0.405	–0.078	–2.63	0.008
Direct Effect	Objective Support → PHQ-9	–0.281	0.109	–0.501	–0.064	–2.58	0.010
	Subjective Support → PHQ-9	–0.207	0.071	–0.351	–0.064	–2.89	0.004
	Support Utilization → PHQ-9	–0.499	0.207	–0.857	–0.052	–2.17	0.030
Total Effect	Objective Support → PHQ-9	–0.408	0.106	–0.623	–0.198	–3.84	<0.001
	Subjective Support → PHQ-9	–0.279	0.073	–0.425	–0.135	–3.81	<0.001
	Support Utilization → PHQ-9	–0.667	0.200	–1.068	–0.279	–3.33	<0.001

SE, standard error. 95% CI, 95% Confidence Interval.

## 4. Discussion

This is the first study with a large sample to invest the mechanism of sex difference in the association between social support and MDD by a mediation analysis with Interoception mediator. Our results showed that depression in female MDD patients was more severe than that in male MDD patients, and the social support in female MDD patients was less than that in male MDD patients. In male MDD patients, the Noticing of Interoception dimension plays a partial mediating role in social support and depression status, but in female MDD patients, the Self-Regulation and Trusting of Interoception dimensions play a partial mediating role in social support and depression status.

In contemporary society, the discourse surrounding the pervasive and multifaceted nature of social pressures faced by women garners substantial academic interest. This examination is rooted in an understanding that, despite significant strides in sex equality and women’s rights, females across diverse cultures and societies continue to navigate a complex web of expectations, roles, and challenges that contribute to heightened levels of social pressure [73, 74]. These pressures emanate from various spheres, including but not limited to, familial obligations, workplace dynamics, societal norms, and cultural practices, interacting to shape the lived experiences of women [75]. The differential impact of social support on male and female MDD patients has emerged as a significant area of inquiry in the fields of psychology, psychiatry, and social sciences [76]. Studies and analyses have increasingly indicated that the disparity in social support received by women and men may contribute to the observed variations in the severity and outcomes of depression among these groups [77, 78, 79]. Consistent with the above findings, our results indicate that female MDD patients have significantly less social support than do male MDD patients, which may be one of the reasons for the more severe depressive symptoms in our female patients.

Nowadays, the imperative of bolstering social support for women emerges as a crucial strategy to mitigate the incidence of MDD. This necessity is underscored by a growing body of evidence indicating a disproportionately high prevalence of MDD among women, attributable to a complex interplay of biological, psychological, and social factors [80]. Enhancing social support for women is not only a matter of addressing an immediate health concern but also a long-term investment in the socio-economic and psychological well-being of society at large [81]. By recognizing the multifaceted causes of MDD among women and implementing comprehensive support systems, we can significantly reduce the incidence of MDD, thereby contributing to a more equitable and healthy society.

In the exploration of the intricate relationship between social support and MDD, many academic inquiries have delved into the mechanisms by which social support influences depressive outcomes. Despite the well-documented association indicating that enhanced social support is inversely related to depression [22, 27, 28], the precise mechanisms underpinning that relationship remain somewhat elusive and complex. Our results confirmed that MDD caused by inadequate or low-quality social support is mediated by interoception; this sheds light on the biological mechanism of MDD that are influenced by poor social support. Our results showed that although interoception mediates the manner in which inadequate or low-quality social support to leads to MDD, there are differences in the interoception-mediating function between male and female MDD patients. This may also be one of the physiological mechanisms for the reason why female MDD patients have more severe depressive symptoms than do male MDD patients. Our findings suggest that to reduce the incidence of MDD in women, we should not only increase social support, but also improve the quality of the social support, i.e., we should provide more social support aimed at improving interoceptive function, such as mindfulness therapy and other technologies.

To summarise, the sex disparity in social support for MDD patients underscores the need for a gender-sensitive lens in both research and treatment paradigms. Understanding and addressing the nuanced ways in which social support operates for men and women with MDD is crucial in devising effective interventions and supports that can mitigate the severity of MDD and enhance recovery outcomes for all individuals, irrespective of sex.

There are two limitations in this study. First, the samples we used were all from the Chinese mainland, so our conclusions are incomplete. Future research should involve large-scale, multi-centre studies that span ethnic, cultural, and regional boundaries, in order to validate our findings. Second, the present research did not extend to the effect of interventions, such as mindfulness therapy, on interoception in MDD. Supplementing a study like this with intervention research will help us further clarify the mediating role of interoception in MDD.

## 5. Conclusions

Our findings indicate that female patients with Major Depressive Disorder receive significantly less social support than do their male counterparts, which contributes to more severe symptoms. The quality and adequacy of social support is crucial due to mediation by interoception, highlighting a biological mechanism behind MDD. Differences in the interoception-mediating role in males and females suggest a physiological reason for the heightened severity of depressive symptoms in females. To mitigate MDD in women, enhancing both the quantity and quality of social support, particularly through methods like mindfulness therapy, that improve interoceptive awareness, is essential.

## Availability of Data and Materials

The datasets used and/or analysed during the current study are available from the corresponding author on reasonable request.
